# An Aluminum Electro-Thermally Actuated Micro-Tweezer: Manufacturing and Characterization

**DOI:** 10.3390/mi14040797

**Published:** 2023-03-31

**Authors:** Rodica-Cristina Voicu, Catalin Tibeica

**Affiliations:** National Institute for Research and Development in Microtechnologies—IMT Bucharest, 126A, Erou Iancu Nicolae Street, 077190 Voluntari, Ilfov, Romania

**Keywords:** micro-tweezer, gripper, electro-thermal, aluminum, V-actuator

## Abstract

In this paper, we present the investigations of an aluminum micro-tweezer designed for micromanipulation applications. It includes design, simulation, fabrication, characterizations, and experimental measurements. Electro-thermo-mechanical FEM-based simulations using COMSOL Multiphysics were performed to describe the behavior of the micro-electro-mechanical system (MEMS) device. The micro-tweezers were fabricated in aluminum, as structural material, by surface micromachining processes. Experimental measurements were performed and compared with the simulation results. A micromanipulation experiment was performed using titanium microbeads from 10–30 µm to confirm the performance of the micro-tweezer. This study serves as further research regarding the using of aluminum as structural material for MEMS devices designated for pick-and-place operations.

## 1. Introduction

In recent decades, micro-electro-mechanical system (MEMS) production has increased significantly. MEM devices offer great potential for micromanipulation applications due to their remarkable features, such as small size, low power consumption, large frequency quality factor, low-cost production, etc. [1,2,3]. As a distinctive type of miniaturization tool, the MEMS-based robotic microgripper has been widely engaged in the manipulation of small micro-objects, material characterizations, and micro assembly. Microgrippers used as end-effectors are critical gears for holding and manipulating delicate entities providing advantages in terms of compact size and low cost. These tools are suitable for a variety of operations, such as handling, pick-and-place, positioning; for biological micro-manipulations, such as cells, blood vessels, and tissues; for applications in the micro assembly of MEMS and MOEMS components (lenses, fibers); in micro-robotics [4,5,6,7,8]. Micro-tweezers are regularly used for the micro-manipulation of micro-objects with dimensions from 1 to 100 µm and reach features of reliable precision, variable applied force, and wide-ranging jaw opening.

Different actuation principles, such as electrostatic [9,10,11], electro-thermal [12,13,14,15,16,17,18,19], piezoelectric [1,5,20,21,22], shape memory alloys [1,23], electro-magnetic [1,24,25,26], optical [27,28] and pneumatic [29], are proposed for the control of the micro-tweezers motion. Piezoelectric and electro-thermal actuations are the most dominant in commercialized micro-tweezers. Electro-thermal micro-actuators are usually components in microsystems and can be power-driven electrically through Joule heating or optically with a laser. The electro-thermal actuation has several advantages, such as higher displacement for lower input voltages, low power consumption, and fast and reliable operation. The factors of force and displacement are more important than other factors influencing the design of the actuators. Their disadvantages include operating at higher temperatures.

Silicon, polymers, and metals have been extensively used as structural materials for MEMS [30,31,32]. Great thermal conductive metals with low costs, such as aluminum (Al) and copper, have been exploited extensively to manufacture micro-heaters or MEMS devices [32,33]. 

Aluminum is the world’s most abundant metal, and this versatility makes it the most widely used metal after steel, having a strength-to-weight ratio superior to steel. Pure aluminum is soft, corrosion resistant, non-toxic, ductile, and has high electrical conductivity. It is widely used for foil and conductor cables; however, alloying with other elements is necessary to provide the higher strengths needed for other applications [34]. Aluminum is one of the lightest engineering metals, but when exposed to air, a layer of aluminum oxide forms almost immediately on the surface, although this layer has excellent resistance to corrosion. It is fairly resistant to most acids but less resistant to alkalis. By exploiting numerous combinations of its valuable properties, such as strength, corrosion resistance, lightness, formability, and recyclability, aluminum is being employed in a continuously growing number of applications. This array of products ranges from structural materials through to thin packaging foils. The thermal conductivity of aluminum is approximately three times greater than that of steel, which makes aluminum an important material for both cooling and heating applications, such as heat-exchangers. Along with copper, aluminum has an electrical conductivity high enough for use as an electrical conductor. Regarding the mechanical properties of aluminum, this metal can be severely deformed without failing. It can also be cast to a high tolerance and can be formed by rolling, extruding, drawing, machining, and other mechanical processes. The tensile strength of pure aluminum is approximately 90 MPa, but this can be increased to over 690 MPa for some heat-treatable alloys [34].

Aluminum films and aluminum alloys are usually chosen for electronic device applications and include most of the interconnections used in the semiconductor chips [35,36]. MEM devices using pure aluminum as the structural material have rarely been reported [33,34,35,36]. Few studies were found in the literature, and most used aluminum alloys or did not report on the manufacturing and experimental tests of the proposed structures. 

A mechanically actuated microgripper with a structure consisting of four arms held together with fingers emerging from a pedestal attached to the shaft where the input force is applied is designed and numerically analyzed using different structural materials, such as aluminum, maraging steel, polycarbonate, VeroWhite, and FullCure 720 [37]. The results were compared, and the best response was found to be aluminum [37].

A modular gripping mechanism for the manipulation of multiple objects was proposed in [31]. The projected microgripper combined traditional machining techniques with MEMS technologies to produce a modular mechanism consisting of a sturdy, compliant aluminum base and replaceable end-effectors. The microgripper base was machined from a 7075-T6 aluminum plate. The strength and elasticity of this material make it a common choice for compliant mechanisms [31]. The microgripper was actuated by a resin-coated multilayer piezoelectric actuator but the tips were fabricated using silicon.

Another paper studied the design process, simulation, and testing of a microgripper that can manipulate and assemble a platinum resistance temperature probe. Operation tests using SolidWorks and ANSYS software were conducted, and the material best-suited was found to be Aluminum alloy 7075-T6 as it could produce a large jaw tip displacement of 0.7 mm without exceeding its tensile yield strength limit [38]. A shape memory alloy was chosen for the actuator to close the microgripper jaws in this case.

An electro-thermally actuated “hot and cold arm” microgripper was analyzed in [39], in which different materials, such as polysilicon, aluminum, and iron, were chosen. The results obtained for aluminum showed better performance than iron and polysilicon in terms of displacement and temperature distribution. The displacement produced when aluminum was chosen was approximately 25 μm and obtained stress was approximately 0.4 GPa, which is less than the tensile strength of aluminum. However, these structures were not fabricated and experimentally analyzed.

Moreover, a chevron electro-thermal actuator was designed and analytically modeled using aluminum as a material for the actuator because of its good electrical and thermal properties [40]. A large displacement was simulated, approximately 10.94 µm, with a lower operating temperature of 448 °C for a single beam [40]. However, again, this proposed chevron was not fabricated. In our previous works [33,41], a MEMS chevron-type thermal actuator and a thermally actuated MEMS switches based on V-shape beams have been designed and fabricated using aluminum as the structural material.

In this work, we investigated an electro-thermally actuated micro-tweezer to be fabricated using pure aluminum as structural material, by surface micromachining processes. Simulation, fabrication, characterization, and experimental tests and measurements were performed. Electro-thermo-mechanical finite element simulations were performed to describe the behavior of the MEMS device using COMSOL Multiphysics software. Experimental investigations were performed and compared with the simulation results. The functionality of the micro-tweezer was confirmed through a micromanipulation/pick-and-place experiment of microparticles. 

The aim of this paper was to study the feasibility of pure aluminum as the structural material for micro-tweezers and as a cheap material to be used for MEMS fabrication with application in micromanipulation domains.

## 2. Materials and Methods

In this section, the micro-tweezer design, materials, and the fabrication process is presented.

### 2.1. Micro-Tweezer Design

The micro-tweezer was designed using the principle of electrically driven thermal actuation based on the V-shaped configuration (chevron shape) that produces translational displacement when heated. There are numerous research works confirming that this kind of configuration offers the advantage that, at a given temperature, the V-shaped structure can deliver a larger tweezer opening [42] in comparison with other configurations usually employed, such as a U-shape structure. Other advantages include the simple structure design, generating a large force, and a lower drive voltage. V-shaped actuators are generally preferred because of their greater performance in liquids. It seems that this configuration is more robust than other types of actuators since the functioning principles of the tweezers driven by V-shaped electro-thermal actuators do not depend on the internal temperature modification [42,43,44,45,46]. 

**Figure 1:**
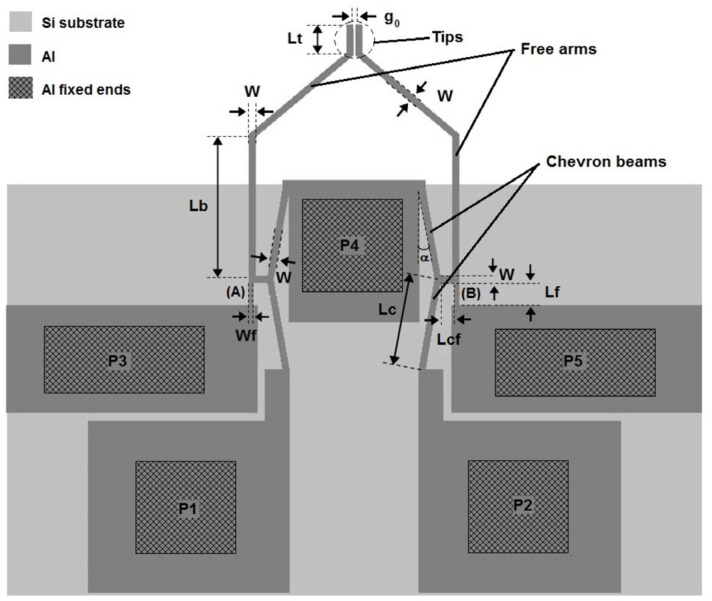
Schematic of the proposed micro-tweezer design: micro-tweezer materials and notations.

Two beams compose the actuator, named “chevron beams”, which are connected to the body of the micro-tweezer at two fixed ends (Figure 1). It is possible to add more pairs of chevron beams to the design, but the dimensions of the micro-tweezer will increase the distances between the free parts of the micro-tweezer. As such, in this work, we have chosen only one chevron pair to study the structural material proposed, which also makes for a compact design. Additionally, a smaller size and less compact or more complicated configuration increases critical technological issues or can cause a tricky release of the actuator parts. The object of this work was to design a monolithic robust micro-tweezer capable of obtaining considerable roto-translational displacements of some of its parts through electro-thermal actuation.

In this study, the micro-tweezer was composed of two pairs of chevron actuators and one actuator consisting of two “chevron beams”, designed with an angle of 10° [33,41,46] and a width of 20 µm (Figure 1). The chevron actuator pairs were connected with the free arms and fixed end parts by thin flexural hinges with a width of 10 µm (Figure 1, (A)-(B)) in order to give the structure higher flexibility. The micro-tweezer was designed to be actuated by applying a voltage between the Al fixed ends, namely the pads P1 and P2 (Figure 1). The pads P3, P4, and P5 were used only to fix the other micro-tweezer parts, namely the chevron beams (P4) and the flexural hinges (P3 and P5).

This kind of design was used before for a SU-8/Au-based micro-tweezer [46] and slightly modified and reprocessed in this study for experimentally analyzing aluminum as the structural material (Table 1).

The designed micro-tweezer had a total length of 930 µm while the free arms and the tips were designed with a width of 20 µm. The micro-tweezer was considered in the normally closed operation mode, with an initial opening of 10 µm. When the micro-tweezer was electro-thermally actuated, the chevron beams expanded, generating a translational displacement, which was utilized to push the free arms of the micro-tweezer further apart, accomplishing an open state and, subsequently, it could grip, pick, and place a micro-object. The normally closed state allows the micro-tweezer to hold and move micro-objects without being continuously heated by the electro-thermal actuation, which is desired to reduce the amount of heat dissipated.

### 2.2. Materials and Fabrication

The micro-tweezer was fabricated using standard micromachining techniques, photolithography, and metal deposition techniques. The process uses two different masks, one for the front side (Mask A) of the wafer to configure the micro-tweezer structures and one for the back side (Mask B) of the wafer used for releasing the chips with structures. The Al films were grown on silicon (100) substrates, 4-inch wafers, after standard chemical cleaning without previous other treatment. The substrate was maintained at room temperature during sample preparation. Aluminum films were deposited by evaporation in a conventional vacuum evaporation system, NEVA EDV-500A Electronic Beam Gun, EVD-500A Basic System. The material used for film preparation was high-purity (99.95–99.99%) Al rod (from Balzers). 

**Figure 2:**
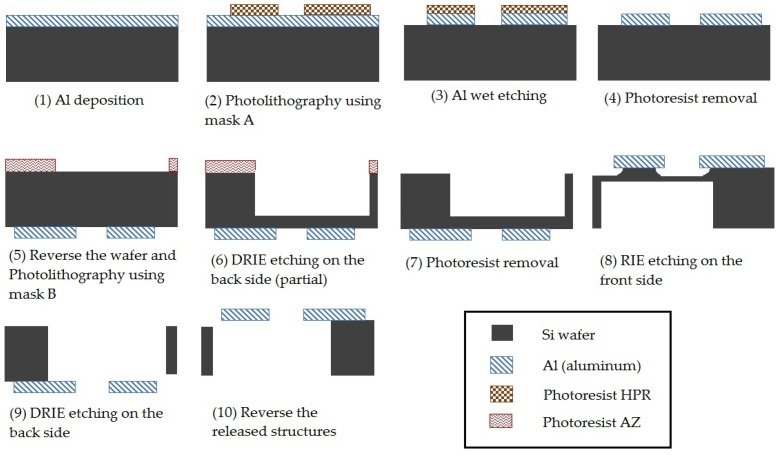
Flowchart of the micro-tweezer fabrication process: (1) Aluminum deposition on the Si substrate; (2) Photolithography using first mask A and HPR photoresist; (3) Aluminum wet etching; (4) HPR photoresist wet removal; (5) Reverse of the wafer and photolithography using AZ photoresist on the back side using second mask B; (6) Partial DRIE etching on the back side of the wafer; (7) AZ photoresist wet removal; (8) Reverse of the wafer and RIE etching on the front side of the wafer to release the structures; (9) Reverse of the wafer and complete DRIE etching of the Si wafer; (10) Reverse of the wafer with released structures and chips.

The fabrication flowchart is shown in Figure 2, and is described as follows:

(1) A layer of aluminum (Al) 2 µm thick is deposited by evaporation on a silicon (Si) <100> wafer in two steps, each a thickness of approximately 1 µm.
(2) A first photolithography process using the first mask (mask A) is conducted with positive photoresist (HPR 402) on the wafer’s front side to shape the Al layer; HPR 402 was spin-coated on the Al layer at 3000 rpm to obtain a thickness of approximately 1.6 µm. A first thermal treatment was realized firstly, at 90 °C for 30 min and a second one, after exposure at 110 °C for 30 min.
(3) The wet etching of Al is conducted from 4–5 min in a solution composed from CH_3_COOH, HNO_3_, and H_3_PO_4_.
(4) The HPR photoresist is removed by acetone.
(5) A second photolithography process using the second mask (mask B) is conducted with positive photoresist (AZ 4562) on the back side of the wafer; the AZ 4562 positive photoresist layer was spin-coated at 3000 rpm to obtain a thickness of around 6 µm; a first thermal treatment was realized at 90 °C for 30 min and a second one, after exposure at 125 °C for 30 min.
(6) The dry etching (DRIE) of Si was performed on the back side of the wafer using SF_6_ gas in order to etch to a depth of around 450 µm.
(7) The photoresist AZ is removed by acetone.
(8) The dry etching–Reactive Ion Etching (RIE) of silicon was performed on the front side of the wafer using an RIE system (Sentech Instruments GmbH, Berlin, Germany) using SF_6_ gas in order to release the aluminum structures from the silicon substrate.
(9) Another dry etching (DRIE) of Si was performed on the back side of the wafer using SF_6_ gas in order to completely release the chips with the released structures.
(10) The chips with released structures were reversed with the back side down to test them.

## 3. Results

In this section, the behavior of the fabricated micro-tweezer is characterized. The electro-thermo-mechanical response, including the openings and the temperatures reached in the micro-tweezer as a function of the applied electrical current, are evaluated. Afterwards, the functionality of the micro-tweezer is confirmed through a micromanipulation of microbeads. 

### 3.1. Dimensional Characterization of the Fabricated Micro-Tweezer

**Figure 3:**
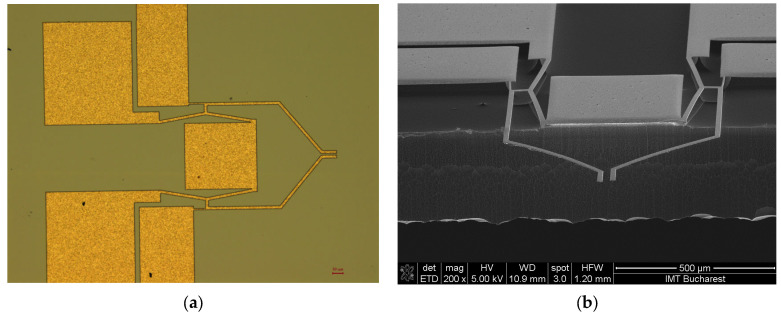
Images of a fabricated micro-tweezer: (**a**) Optical image before release; (**b**) SEM image after release.

**Figure 4:**
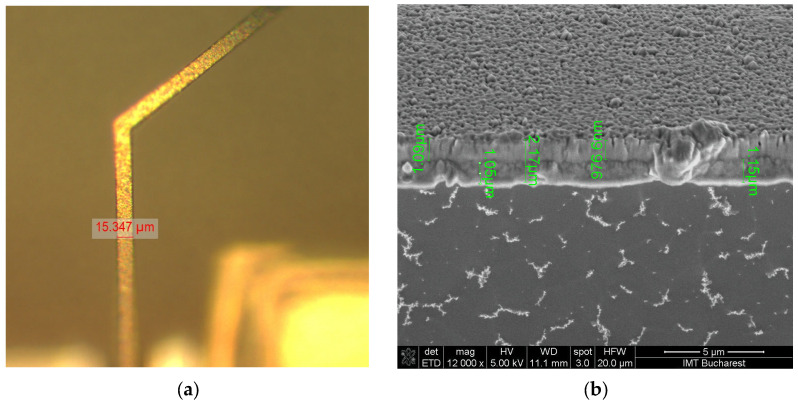
Measured dimensions of the fabricated micro-tweezer: (**a**) Optical measurement of the arms’ width after release; (**b**) SEM image of thickness measurements.

Different characterization methods were performed in order to analyze the fabricated micro-tweezer. First, the fabricated micro-tweezer was inspected before release using an optical microscope and after release using a SEM (Figure 3). The optical images taken before the release step shows a well-configured shape of the micro-tweezer components. Second, the geometrical dimensions were measured using the optical microscope and the Al layer thickness using SEM for the released structure. All the widths of the structure were found to be smaller than they were designed. For example, the measured width of the free arms, tips, and of the chevron beams (W in Table 1) was approximately 15.3 µm instead of 20 µm. Similarly, the width of the flexural hinges (Wf in Table 1) was evaluated at 5 µm. Further, the thickness (t in Table 1) of the Al layer was found to be larger than designed, approximately 2.2 µm instead of 2 µm (Figure 4). The distance between the tips, or the initial opening (g_0_ in Table 1), was estimated at ~14 µm. Hereinafter, any modification of the distance between the tips due to actuation, will be referred as opening displacement, g–g_0_, where g is the total opening between the tips after actuation.

### 3.2. Actuation Tests of the Micro-Tweezer

**Figure 5:**
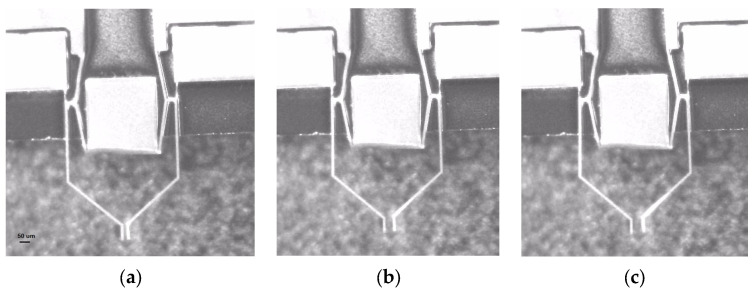
Micro-tweezer functional tests: (**a**) initial normally close state, at 0 mA; (**b**) open state at 160 mA; (**c**) for the last opening state after the actuation at 170 mA.

Different micro-tweezers were prepared for the experimental characterization by manually fixing the released chips on a silicon substrate. Experimental tests were performed to analyze the in-plane displacements of each micro-tweezer’s arm when it was electrically actuated (Figure 5). A controllable DC power supply was used to apply voltages to the actuation pads. Electrical connections between the power supply and the contact pads of the structure were established using two probe station manipulators, each having a 3-axis positioning system. The in-plane movements were monitored with an optical microscope and a video camera (DinoLite). The micro-tweezer was actuated by applying an electrical voltage ranging from 0 to 1 V and measuring the electrical current in steps of 10 mA. The time interval between each step was more than 5 s to ensure that steady-state conditions were achieved. At each actuation step, an image was acquired for later measurements of the displacements. Important facts to get accurate measurements using the optical images are the lighting and the position of the micro-tweezer and the camera to minimize the shadows.

A maximum opening displacement, g-g_0_, of 22 µm (or 36 µm total opening, g) was measured at an input current of 160 mA. By further increasing the current, out-of-plane displacements of the movable parts started to occur, making the operation and measurements unreliable. However, at an input current of 170 mA, we still managed to measure an opening displacement of 27 µm (41 µm total opening), and at 180 mA input current, the measured opening displacement was approximately 33 µm (47 µm total opening). The relation between the input current (up to 160 mA) and the micro-tweezer’s opening displacement, both measured and simulated, is graphically represented in the next Section.

### 3.3. Characterization by FEM Simulations

**Figure 6:**
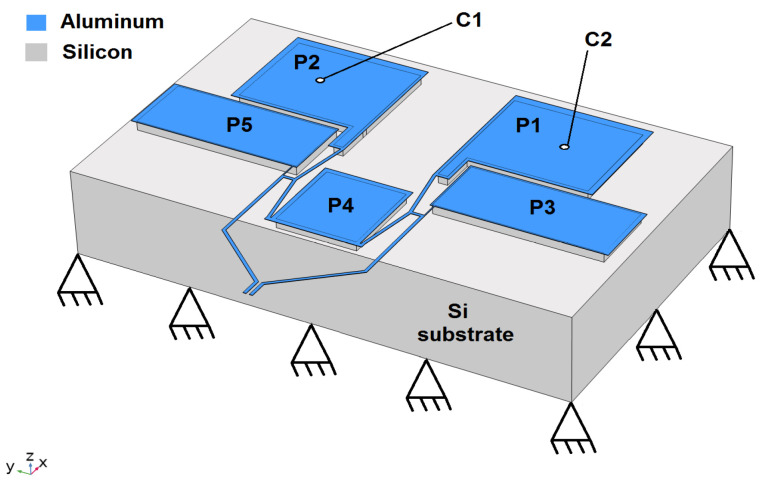
3D model of the designed micro-tweezer (COMSOL Multiphysics) with the surfaces where boundary conditions were set: bottom surface of Si substrate-mechanically fixed and temperature constraint at T_0_; C1 and C2–electrical contacts; top faces (both Al and Si)-natural convection.

For numerical simulations, a virtual model was developed using COMSOL Multiphysics software. A three-dimensional (3D) model of the micro-tweezer is shown in Figure 6. 

The mechanical, thermal, and electrical properties of the materials involved, as well as the loads and boundary conditions, were set in the model such as to emulate as much as possible the real device. To numerically simulate the electro-thermo-mechanical behavior of the designed micro-tweezer in COMSOL Multiphysics, the Solid Mechanics, Heat Transfer in Solids, and Electric Currents interfaces were used in conjunction with Thermal Expansion and Electromagnetic Heating interfaces for multiphysics couplings. The required boundary conditions for each of the physics interfaces were set as follows:

- for the mechanical problem, the bottom face of the silicon chip is considered fixed;
- for the thermal problem, natural convection was set on the top faces of the silicon chip and aluminum structure, and fixed temperature (ambient at T_0_ = 20 °C) on the bottom face of the silicon, as well as on electrical contacts (C1 and C2) on pads P1 and P2, respectively;
- for the electrical problem, the input current (Ic) on the electrical contact C1, and the output current (-Ic) on the electrical contact C2, were set. The C1 and C2 circular areas replicate the contacts of the electrical probes on the chip.

Symmetry boundary conditions were conveniently exploited to reduce the computational costs, so only one-half of the micro-tweezer has been considered for the simulations.

The material properties for aluminum were chosen from the literature, measured and/or from the COMSOL material library (Table 2). Because the Young’s modulus, thermal conductivity, and electrical resistivity strongly depend on the temperature, they were introduced as the function of temperature [47,48,49,50]. Silicon properties were chosen from the COMSOL Material library [50].

The Young’s modulus of aluminum as the function of temperature (*T*), *E*, was considered using Equation (1) [49]:

(1) $$E\left(T\right)=-3.9\times e^{-0.0033\times T}+79$$

The electrical resistivity of aluminum as the function of temperature (*T*) follows Equation (2) [50]:

(2) $$\rho \left(T\right)=\rho_{0}\left(1+\alpha \left(T-T_{0}\right)\right)$$

where $\rho_{0}$ is the electrical resistivity of aluminum at *T*_0_, the room temperature, and α is the resistivity temperature coefficient.

**Figure 7:**
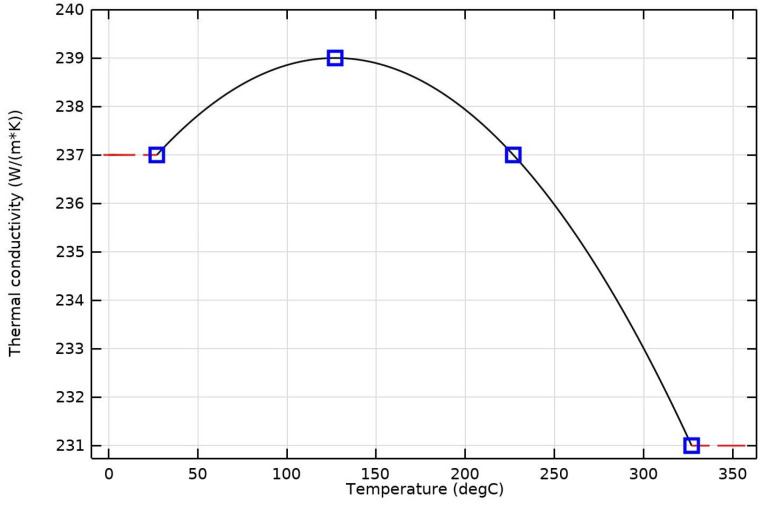
Interpolated values for aluminum thermal conductivity using cubic spline.

For the thermal conductivity of aluminum as the function of temperature, some values from the literature [49] were interpolated and the graph is shown in Figure 7.

The micro-tweezers were designed considering the properties of the materials involved, the technological limitations, and the area of applications. By means of FEM simulations, the electro-thermo-mechanical behaviors were initially estimated on the virtual models of the micro-tweezers. However, the manufactured devices showed relatively large discrepancies as opposed to the simulations. We tried to figure out where the source of errors came from. First, the electro-thermo-mechanical behavior is sensitive to dimensional variations, especially the dimensions of the chevron arms. From electrical point of view, the length, width, and thickness of the arms, and the actual value of Al resistivity, affect the electrical resistance and, consequently, the thermal and mechanical behavior. By experiments, we were able to measure the real geometric features of the manufactured structures, and the electro-mechanical behavior was determined. The last included the current–voltage curve and the mechanical displacement of the tweezers’ arms as function of applied voltage or current.

The thermal behavior, in the absence of direct temperature measurement methods, can also be indirectly evaluated with the help of simulation, but it requires a good model for the device. With this aim, we managed to measure the actual electrical resistivity of the Al layer by two different methods. The first one was the four-probe method, which is the primary technique in measuring the sheet resistance of a material, while for the second one, a resistor with precise dimensions, was configured in the deposited Al layer, and its electrical resistance was measured by a multimeter. Both the methods gave a consistent value of 4.35 × 10^−8^ Ω∙m for the resistivity, instead of 2.64 × 10^−8^ Ω∙m, which is supposed to be for pure aluminum at room temperature. As a possible explanation for discrepancy is that the difference could be due to the roughness of the deposited Al layer [36], which was higher than expected from a standard process.

Once the input data in the virtual model were updated, a series of simulations were performed, and the results were compared with the measurements. The other material properties of aluminum were taken from the literature, as listed in Table 2. Other critical settings in the numerical simulations included the proper boundary conditions, especially for the thermal problem. The value for the natural convection coefficient at the microscale largely differs from the macroscale [51]. From the literature and from our own experience, a good assessment considering the scale of the investigated device is 2000 W/(m^2^∙K), observing that 10% variation around it does not impact too much on simulation results. Additionally, the heat transport through the conduction mechanism was carefully included in the model by considering all thermal contacts involved in the experiments, namely the chip on its support and the electrical wiring on the pads (C1 and C2 in Figure 6).

**Figure 8:**
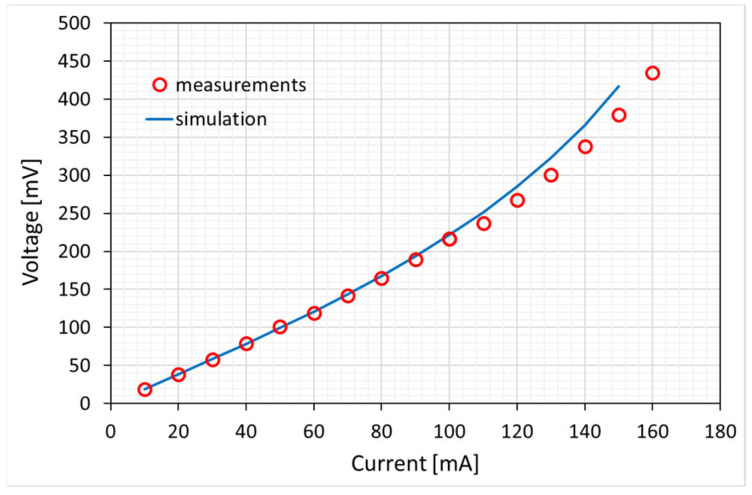
Voltage vs. current from measurements and simulation.

The results show a quite a good match between the simulation and measurements in the case of electrical behavior (Figure 8). 

The slightly increasing difference between the curves at higher currents was most probably related to the resistivity temperature coefficient of aluminum, the value of which was taken from the literature and not measured for our samples.

**Figure 9:**
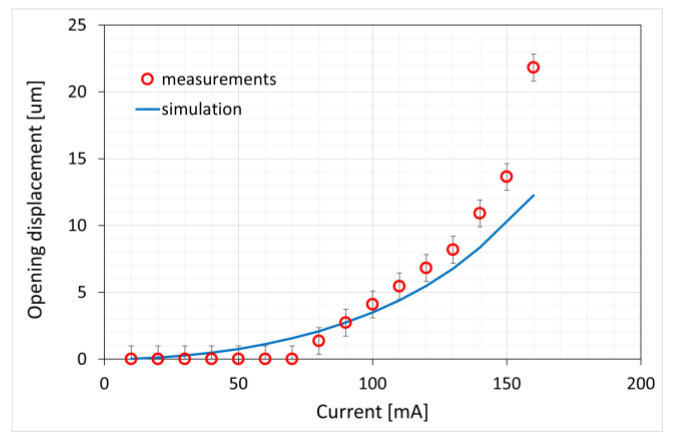
Measured and simulated displacement opening, g–g_0_, as function of input current. The error bars are ±1.5 µm.

Regarding the mechanical behavior, the current-controlled movement of the micro-tweezer’s arms was dependent on the coefficient of thermal expansion (CTE) of the real material. In the absence of a measured value for CTE, the value we used was from the literature (see Table 2 and [47]). The measured and simulated displacements of the micro-tweezer’s arms as function of input current are shown in Figure 9. Obviously, the numerical simulations underestimate the displacements, but there are the parameters with guessed values at stake, such as Young’s modulus and CTE.

The thermal behavior includes the temperature map of the micro-tweezer, which is also dependent on current. The maximum temperature occurs in the chevron arms, but due to the high thermal conductivity of aluminum, the micro-tweezer’s arms might also reach quite high temperatures in operation. 

**Figure 10:**
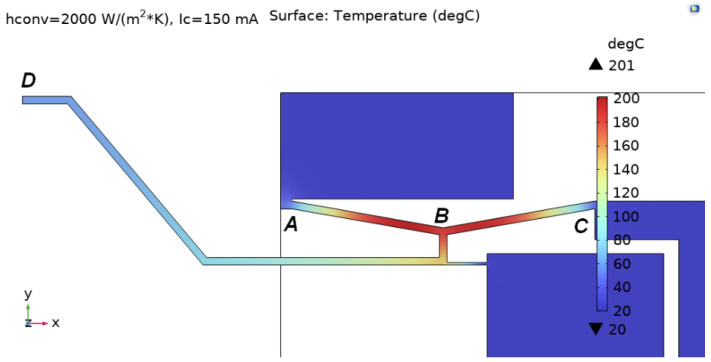
Temperature map of the micro-tweezer at 150 mA input current.

**Figure 11:**
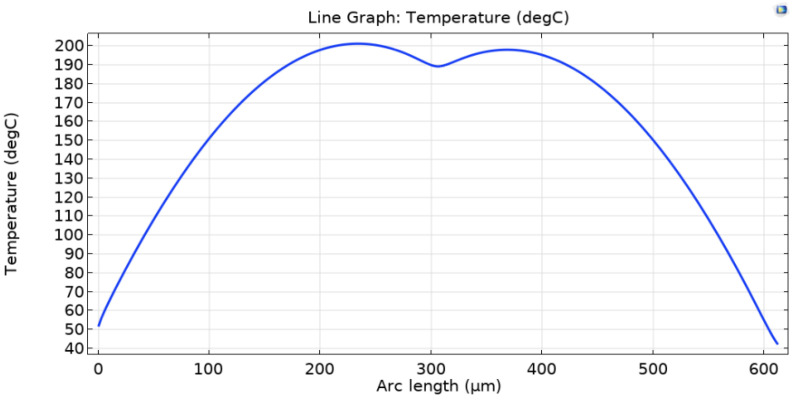
Temperature profile along the A-B-C path (Figure 10) of the Chevron arms at 150 mA input current.

A direct measurement of temperature distribution in the real device was not possible at this stage of our work; however, based on the current–voltage curve (Figure 8), an average temperature of the chevron arms could be derived by treating them as lumped elements with a known temperature-dependent resistivity. This required assuming that temperature-dependent resistance of the entire device is only due to the chevron part of the circuit, which acts as a heater. However, we used the numerical simulations to determine the temperature profile along the heated arms (Figure 10 and Figure 11).

**Figure 12:**
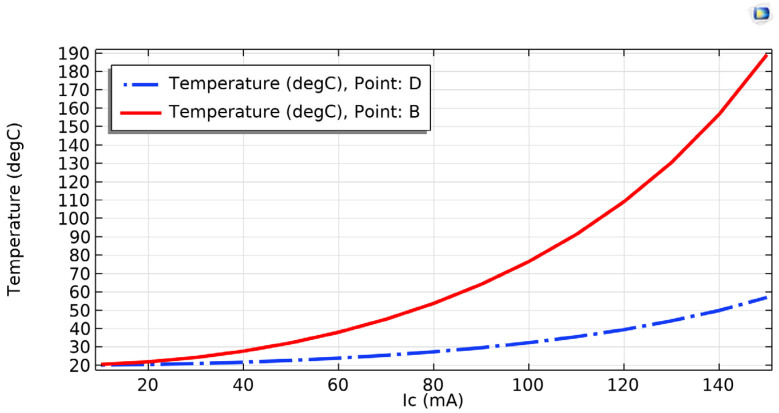
Temperature at points B and D as function of input current (Ic).

Due to the connection beam with the hinge at point B, there is a small dip in the temperature profile, shown in Figure 11, but close to the maximum temperature reached by the structure. For practical purposes, it was also important to predict the temperature at point D, where the picked object would be contacted. Figure 12 shows the simulated temperatures reached at points B and D (referred to in Figure 10) as function of input current.

### 3.4. Functional Tests of the Micro-Tweezer

**Figure 13:**
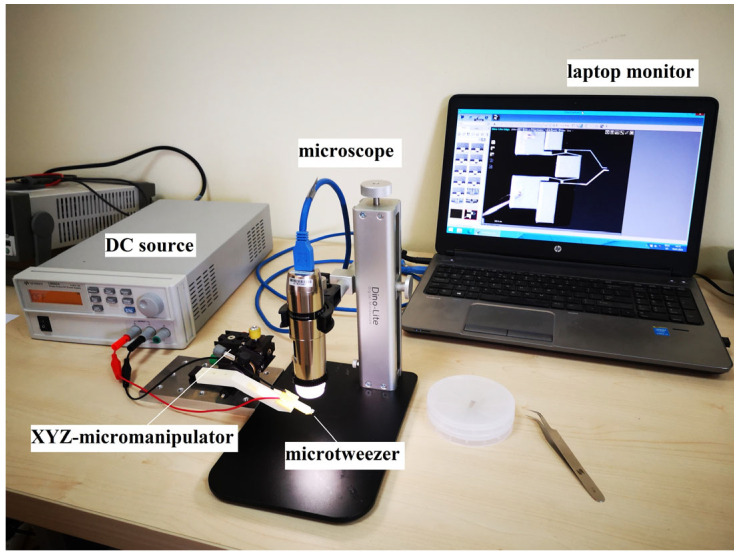
The experimental setup for micromanipulation applications.

To confirm the performance of the micro-tweezer in micromanipulation applications, a pick-and-place experiment on titanium microbeads from 10–30 μm in diameter was performed. The micro-tweezer chips were mounted on a PCB (printed circuit board) plate and gold wires were connected from the aluminum fixed end pads of the micro-tweezer to the PCB electrical routes. The PCB plate with a fixed micro-tweezer was attached on a polymeric arm that was attached to an XYZ-micromanipulator. After that, electrical wires were fixed between the PCB electrical routes and DC controllable electrical source. The electrical wires were then connected to the DC source. The position of the micro-tweezer allowed the initiation of the open/close gripping motion at any instance during the experiment. A digital microscope Dino-Lite with dedicated software and a laptop were assembled with the XYZ-micromanipulator and the DC source to complete the functional tests (Figure 13).

**Figure 14:**
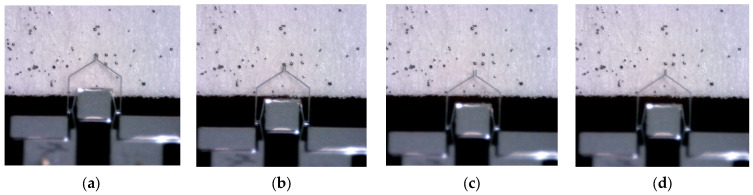
Micromanipulation of microbeads: (**a**) Pick a microbead from the first arranged line; (**b**) Place the microbead on the second line, under the first line; (**c**) Release the microbead; (**d**) Close the micro-tweezer tips.

The titanium microbeads were placed on a polymeric plate fixed on a stationary holder under the digital microscope. Figure 14 demonstrates the results of the pick-and-place experiment. First, the micro-tweezer, in a closed state with no actuation, was placed near a microbead using the XYZ-micromanipulator arm. Next, the micro-tweezer was switched to an open state by controlling the DC source and was positioned to have the microbead between the micro-tweezer tips. Consequently, the micro-tweezer tips were closed by switching off the electrical current or decreasing the electrical current value such that the microbead was firmly held between the tips. After that, the XYZ-micromanipulator arm was moved to another place to release the microbead. The operations were repeated to place the microbeads on the polymeric plate and one and two rows with microbeads were formed on the plate.

The successful manipulation of the microbead demonstrated the potential of the proposed micro-tweezer to be used in micromanipulation and pick-and-place applications.

**Figure 15:**
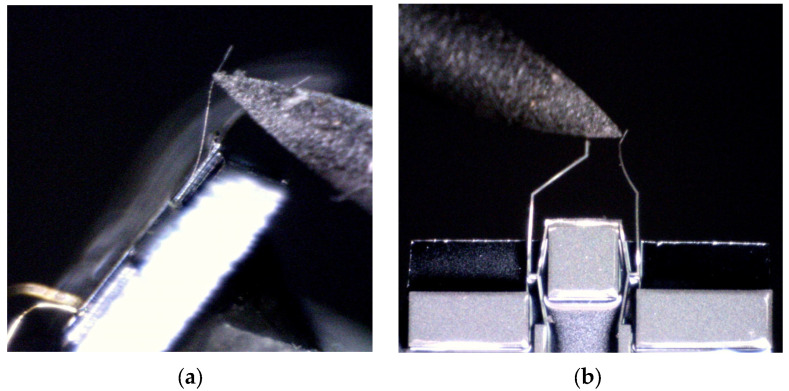
Micro-tweezer tests at forced deformation: (**a**) Bending up of a micro-tweezer free arm; (**b**) Lateral bending of a micro-tweezer free arm.

To test the robustness of the micro-tweezer, a metallic needle was used to apply a force to the free micro-tweezer’s arms. The test was performed by pushing the free micro-tweezer’s arm towards in-plane and out-of-plane directions (Figure 15). The optical inspection showed that the arms remained flexible and recovered to almost the same initial state.

## 4. Discussion and Conclusions

An electro-thermally actuated micro-tweezer using aluminum as the structural material was developed and studied. At the design stage, numerical simulations were per-formed, but manufacturing the real devices revealed dimensional discrepancies with the design, and the performed measurements showed different values for some material properties from what was initially considered. For example, the measured electrical resistivity of the aluminum layer was far from the values reported in the literature for pure aluminum.

However, re-running the simulations on a model with corrected (measured) dimensions, as a post-manufacturing step, helped us to understand what the critical parameters were and how they acted. These last simulation results are presented in this study and compared to the measured ones. Moreover, we performed the new simulations with the aim of estimating some behaviors that could not be directly measured, such as the temperature map of the device.

The results obtained from the finite element simulations in this study are slightly different from the experimental results, and this can be attributed to the following:

- The construction of the virtual 3D model neglected some features occurring in the fabrication, such as the etching profiles and non-uniformity of the deposition of the aluminum on the Si substrate.
- It was not possible to measure all the aluminum properties, such as the coefficient of thermal expansion, resistivity temperature coefficient, or the residual stresses.
- Displacement measurement errors given by the optical limitations.

Considering the achieved results, further developments should include a more controlled deposition processes, as well as configuring the deposited layer by using the lift-off process, since the dimensions were strongly affected by the etching. The design optimization should also be considered for better control of movement.

In conclusion, this article proposes a robust micro-tweezer to function as a pick-and-place operator of microparts with varied sizes. Its normally closed state enables handling micro-objects in the range from 10 μm to about 50 μm. The micro-tweezer was fabricated by surface micromachining processes using pure aluminum as the structural material and the detailed manufacturing methodology is described. Additionally, numerical simulations were performed, which helped in identifying critical design parameters. Testing of the micro-tweezer included its electrical and mechanical behaviors and confirmed the suitability of the design and the robustness of the mechanical structure made of aluminum. Functional tests demonstrated the ability to pick and release micro-objects in a controlled manner with good reliability.

## Figures and Tables

**Table 1:** Nominal designed geometrical parameters of the micro-tweezer.

Parameter	Value
Distance between the tips, initial opening, g_0_ (µm)	10
Length of the tips, Lt (µm)	90
Width of the free arms, tips, and chevron beams, W (µm)	20
Width of the flexural hinges, Wf (µm)	10
Length of the flexural hinges, Lf (µm)	78
Length of one chevron beam, Lc (µm)	303
Length between the chevron beam and the flexural hinges, Lcf (µm)	40
Length of the free arm before it is bent, Lb (µm)	460
Angle of the chevron beams, α (^o^)	10
Aluminum thickness, t (µm)	2

**Table 2:** Material properties used for FEM simulations.

Property	Value		Unit
	Aluminum	Silicon	
Density	2700	2329	kg/m^3^
Young’s modulus, *E*	See Equation (1)	170 × 10^9^	Pa
Poisson’s ratio, ν	0.35	0.28	
Coefficient of thermal expansion, *CTE*	23.1 × 10^−6^	2.6 × 10^−6^	1/K
Heat capacity at constant pressure, *Cp*	904	700	J/(kg·K)
Thermal conductivity	See Figure 7	130	W/(m·K)
Electrical resistivity at *T*_0_, $\rho_{0}$	4.35 × 10^−8^	-	Ω·m
Resistivity temperature coefficient, α	0.0038	-	1/K

## Data Availability

The data are presented as graphs and tables in the manuscript.
